# Diagnostic value of Video Electroencephalography combined with Magnetic Resonance Imaging-diffusion tensor imaging in epilepsy

**DOI:** 10.12669/pjms.40.4.8858

**Published:** 2024

**Authors:** Lan Wang, Min Fang, Bin Zhu, Xiaoling Wang, Xiang Li

**Affiliations:** 1 Lan Wang, Department of Radiology, Quzhou Hospital of Zhejiang Medical and Health Group, Quzhou, Zhejiang Province 324000, P.R. China; 2 Min Fang, Department of Radiology, Quzhou Hospital of Zhejiang Medical and Health Group, Quzhou, Zhejiang Province 324000, P.R. China; 3 Bin Zhu, Department of Radiology, Quzhou Hospital of Zhejiang Medical and Health Group, Quzhou, Zhejiang Province 324000, P.R. China; 4 Xiaoling Wang, Department of Radiology, Quzhou Hospital of Zhejiang Medical and Health Group, Quzhou, Zhejiang Province 324000, P.R. China; 5 Xiang Li, Department of Neurosurgery, Quzhou People’s Hospital, 100 Minjiang Avenue, Quzhou, Zhejiang Province 324000, P.R. China

**Keywords:** Video electroencephalography, Magnetic resonance imaging, Diffusion tensor imaging, Epilepsy

## Abstract

**Objective:**

To explore the diagnostic value of video electroencephalography (VEEG) combined with magnetic resonance imaging diffusion tensor imaging (MRI-DTI) in epilepsy.

**Methods:**

In this retrospective observational study, clinical data of 60 patients who underwent both VEEG and MRI examinations at the Neurosurgery Department of Quzhou People’s Hospital from December 2020 to March 2023 were retrospectively reviewed, and a total of 55 patients were finally included. We evaluated the diagnostic value of combining VEEG and MRI to determine seizure type, location of epileptic focus, and structural abnormalities of brain tissue, using surgical and pathological results as the gold standard.

**Results:**

The accuracy of the combined approach for determining the seizure type was 98.18%, which was higher than the accuracy of MRI (85.45%, *P*<0.05) or VEEG (81.82%, *P*<0.05) alone. The accuracy of joint examination for lesion location was 100.00%, which was higher than those of MRI (85.45%, *P*<0.05) or VEEG (83.64%, *P*<0.05) alone. Similar abnormal brain tissue structure detection rates was found for both MRI and VEEG (*P*>0.05).

**Conclusions:**

The application of MRI-DTI combined with VEEG to diagnose patients with epilepsy allows for the identification of abnormal structural changes in brain and the location of lesions. Combining both approaches can improve the diagnostic accuracy of each technique alone and provide a reference for the formulation and adjustment of disease management plans.

## INTRODUCTION

Epilepsy is a common chronic neurological disease characterized by a spontaneous recurrence of unprovoked seizures with complex pathogenic factors^1^prognostic counselling and management of the epilepsies. Indeed, the aetiology can be important for determining the recurrence risk of single seizures and so for making a diagnosis of epilepsy. Here, we divide the aetiologies into six categories: structural, genetic, infectious, metabolic, immune (all of which are part of the International League Against Epilepsy [ILAE] classification system that have not been fully elucidated, but are mainly caused by the abnormal discharge of highly synchronized brain neurons.^1^,^2^prognostic counselling and management of the epilepsies. Indeed, the aetiology can be important for determining the recurrence risk of single seizures and so for making a diagnosis of epilepsy. Here, we divide the aetiologies into six categories: structural, genetic, infectious, metabolic, immune (all of which are part of the International League Against Epilepsy [ILAE] classification system These abnormal discharges rely on the abnormal transmembrane movement of ions inside and outside of the cell membrane, and the transient paroxysmal events can be characterized by localized rigidity and movement repetition. Epilepsy endangers the physical and mental health and quality of life of patients, and significantly increases their financial and medical burden.^3^,^4^

Research has shown that individuals with epilepsy and seizures can present structural abnormalities in the brain.^4^ Epilepsy is associated with structural and functional changes in the hippocampus, frontal cortex, temporal cortex, amygdala, and olfactory cortex.^5^ It could be categorized into two main types: generalized epilepsy and focal epilepsy, each of which has many different subtypes.^6^ Widespread patterns of altered subcortical volume and reduced cortical grey matter thickness could be observed in patients with epilepsy.^7^ Most clinical interventions for patients with epilepsy are pharmacological, and commonly used drugs include lamotrigine, phenytoin sodium, and carbamazepine.^8^ Clinicians need to consider the types and characteristics of the disease outbreaks to avoid misdiagnoses and mistreatment during the formulation of treatment plans.^8^,^9^ Therefore, accurate assessments of brain injury in patients with epilepsy are of great significance.^4^,^8^,^9^

Video electroencephalography (VEEG) is commonly used in the diagnosis and treatment of epilepsy and it has its complications. It can record the spontaneous and rhythmic activities of brain cells and evaluate the brain functional status based on the rhythm and waveform of the electroencephalography (EEG) waves. VEEG is simple to operate, reproducible, and low costs, and can show the initiation and transmission processes of disease during an outbreak. However, clinicians cannot easily identify the exact location and range of lesions using VEEG, and its localization specificity and sensitivity are poor.^10^ Magnetic resonance imaging diffusion tensor imaging (MRI-DTI) is a commonly used imaging diagnostic technique to non-invasively detect the distribution characteristics of protons in the brain to paint a structural image. The technique results in clear images without radiation exposure.^11^,^12^ Studies have investigated VEEG combined with functional MRI (fMRI)^13^,^14^, or DTI combined with functional MRI^15^, but few on VEEG plus MRI-DTI. Therefore, we aimed to retrospectively analyze the clinical data of 55 patients with epilepsy in our hospital to assess the diagnostic value of VEEG combined with MRI-DTI for epilepsy.

## METHODS

In this retrospective observational study, clinical data of 60 patients with epilepsy who underwent both VEEG and MRI examinations at the Neurosurgery Department of Quzhou People’s Hospital from December 2020 to March 2023 were retrospectively reviewed, and a total of 55 patients were finally included.

### Ethical Approval

The ethics committee of Quzhou People’s Hospital approved this study on July 2^nd^ 2023, No. 2023-11. Informed consents were obtained from the patients.

### Inclusion criteria:

- Patients with seizure history and multiple evoked standardized electroencephalography test results meeting the epilepsy-related diagnostic criteria.^3^
- Patients who underwent both brain computer and MRI-DTI examinations.
- Individuals older than 18 years.
- Patients undergoing elective surgical treatment within one month after the initial examination.

### Exclusion criteria:

- Patients with symptoms of pseudo seizure and febrile convulsions.
- Patients with intracranial tumor or cerebrovascular disease.
- Patients with systemic diseases that cannot be effectively controlled.
- Patients with claustrophobia, metal implants, or other MRI contraindications.
- Patients with incomplete clinical data.

### VEEG

**Fig.1:**
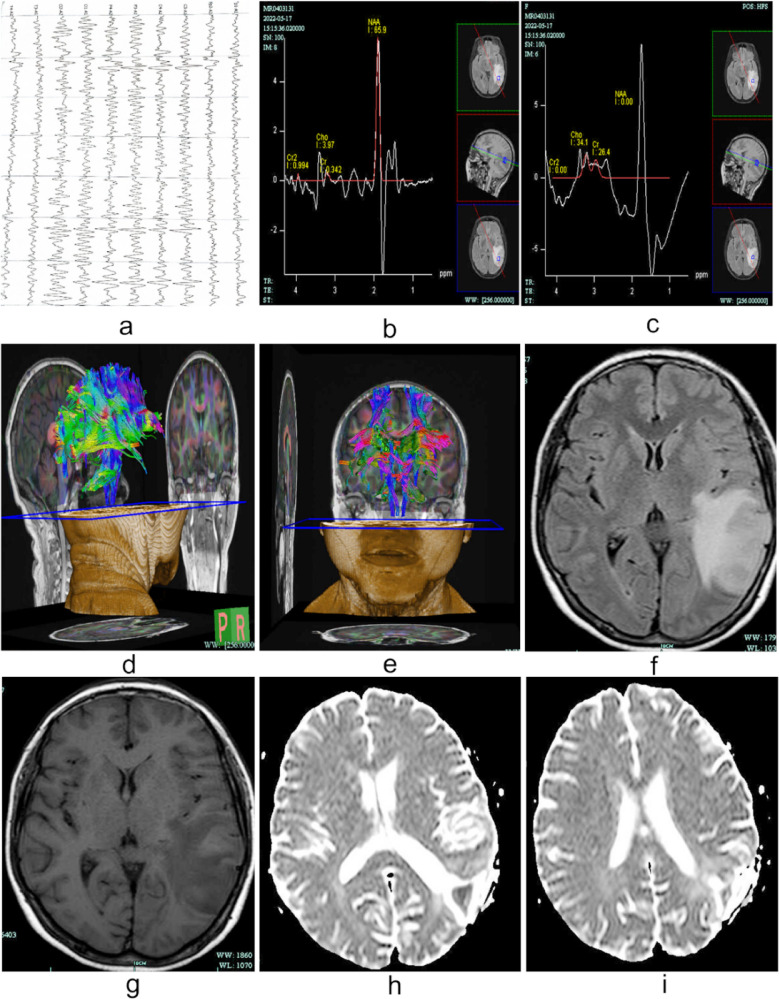
Female, 56 years old. a) VEEG image, which shows a small number of single scattered issuances of sharp and slow waves around 3.0 HZ on the left side, which is diagnostically accurate; b to i) MRI-DTI images: b and c shows a decreased NAA and an elevated Cho peak in the left temporoparietal lesion; d and e shows the left temporoparietal lesion area has disrupted and terminated nerve fibre bundles and nudged peripheral nerve bundles, a slightly high signal shadow of the lesion is visible on DWI (at high b values), which corresponds to an equal low signal on ADC; f and g shows the left occipital lobe is seen as a T2W1, FLIIR high signal, T1W1 low signal shadow with clear borders, high signal on DWI at high b values; h and I shows low signal on ADC

SOLAR2848B/ROVER (Solar Electronic Technologies Co., Ltd, Beijing, China) was used for digital electroencephalography. According to the 10/20 system, the electrodes were placed on the patient’s scalp, with two ear electrodes as reference electrodes.^16^ We applied conductive paste to the surface of the electrodes for fixation and adding bandages or mesh elastic caps to prevent detachment. The parameters were set as follows: transport speed, 30mm/s; digital/analog signal conversion resolution, 24 bits; time constant, 0.3 seconds; and, high frequency filtering, 30 Hz. We allowed the built-in computer system of the device to adjust the graphic signal, ensuring real-time synchronization between the image signal and the VEEG monitoring data, and implemented descriptive recording (3-5 minutes per occurrence). The patients were continuously monitored for 24 hours and completed opening-closing of the eyes test, hyperventilation test, photic stimulation, and sleep monitoring while awake. The seizure type and brain damage location detection are shown in Fig.1-A.

### MRI-DTI

We used Vida and Skyra 1.5T magnetic resonance scanners (MAGNETOM, Siemens, Erlangen, Germany) for head magnetic resonance scanning examinations. We programmed the following settings: proton resonance frequency, up to 200 MHz using standard head coils; routine axial and sagittal scans; FSE sequence T2 weighted scans; SE sequence T1 weighted scans; and transverse T1 and sagittal T2 flair scans. For temporal lobe epilepsy, we increased the spectral analysis scan of both hippocampi.^17^ We used the Siemens Tissue 4D post-processing software to process relevant images, implement iterative reconstructions, and obtain 3D stereo images. Fig.I B-I.

Two specialized physicians with a working experience of more than 10 years in the department analyzed all test results, which we classified as 1) seizure type diagnoses; 2) epileptic lesions location determination; and, 3) abnormal brain structure detection.

### Statistical Analysis

Data were analyzed using SPSS version 20.0 (IBM, Armonk, NY, USA). We expressed categorical variables as frequencies and percentages. Chi-squared test was used to assess differences between ECG and MRI-DTI results. *P*-values lower than 0.05 was considered as statistically significant. All reported *p*-values are bilateral.

## RESULTS

We included imaging and clinical data from 55 patients, including 32 men and 23 women ranging in age between 23 and 71 years (mean age, 47.87±11.02 years). The etiology was clear in 30 patients (two cases of meningitis, seven cases of craniocerebral surgery, five cases of encephalitis, seven cases of postnatal intracranial hemorrhage, two cases of asphyxia, seven cases of premature delivery), and the etiologies for 25 cases were unknown. The accuracy of the combined examination was 98.18%, higher than the separate accuracies of MRI-DTI (85.45%) and VEEG (81.82%) for individual diagnoses, with statistically significant differences (*p*<0.05; Table-I).

**Table-I:** Individual and combined diagnostic accuracies for type of seizure with MRI-DTI and VEEG.

Inspection method	n	Generalized tonic clonic seizure	Absentia attack	Myoclonic seizures	Compound seizure	Complex partial seizures	Partial secondary generalized tonic clonic seizures	Simple partial seizure	Diagnostic accuracy
MRI-DTI	55	15 (27.27)	4 (7.27)	4 (7.27)	3 (5.45)	11 (20.00)	7 (12.73)	3 (5.45)	47 (85.45)
VEEG	55	14 (25.45)	3 (5.45)	5 (9.09)	3 (5.45)	10 (18.18)	6 (10.91)	4 (7.27)	45 (81.82)
Combined diagnostic	55	21 (38.18)	5 (9.09)	3 (5.45)	2 (3.64)	12 (21.82)	5 (9.09)	6 (10.91)	54 (98.18)
*χ^2^_1_/P_1_*									4.356/0.037
*χ^2^_2_/P_2_*									6.465/0.011

***Note:*** χ^2^_1_/P_1_ is a comparison between MRI-DTI and joint diagnosis; χ^2^_2_/P_2_ is a comparison between VEEG and joint diagnosis.

The accuracy of the combined examination was 100.00%, higher than those of MRI-DTI (85.45%) and VEEG (83.64%) for individual diagnoses with statistically significant differences (*P*<0.05; Table-II). Both MRI-DTI and VEEG can detect brain tissue damage in patients with epilepsy, including features such as abnormal neuronal migration, tuberous sclerosis, abnormal signals, brain dysplasia, brain malformations, encephalomalacia, and cerebral atrophy. We found similar brain lesion detection accuracies for MRI-DTI and VEEG (*P*>0.05; Table-III).

**Table-II:** Individual and combined diagnostic accuracies for epileptic focus location with MRI-DTI and VEEG.

Inspection method	n	Frontal lobe	Temporal lobe	Occipital lobe	Parietal lobe	Multifocal	Abnormal whole brain structure	Accuracy
MRI-DTI	55	7 (12.73)	4 (7.27)	2 (3.64)	3 (5.45)	26 (47.27)	5 (9.09)	47 (85.45)
VEEG	55	6 (10.91)	5 (9.09)	3 (5.45)	2 (3.64)	25 (45.45)	6 (10.91)	46 (83.64)
Combined diagnostic	55	6 (10.91)	3 (5.45)	3 (5.45)	2 (3.64)	34 (61.82)	7 (12.73)	55 (100.00)
*χ^2^_1_/P_1_*								6.605/0.010
*χ^2^_2_/P_2_*								7.745/0.005

***Note:*** χ^2^_1_/P_1_ is a comparison between MRI-DTI and joint diagnosis; χ^2^_2_/P_2_ is a comparison between VEEG and joint diagnosis.

**Table-III:** Abnormal brain tissue structure detection accuracies for MRI-DTI and VEEG.

Inspection method	Cerebral atrophy (n=14)	Brain softening (n=16)	Brain malformation (n=2)	Brain Dysplasia (n=17)	Signal abnormality (n=5)	Nodular sclerosis (n=7)	Abnormal neuronal migration (n=6)
MRI-DTI	92.86% (13/14)	100.00% (16/16)	100.00% (2/2)	94.12% (16/17)	100.00% (5/5)	100.00% (7/7)	100.00% (6/6)
VEEG	85.71% (12/14)	87.50% (14/16)	100.00% (2/2)	82.35% (14/17)	100.00% (5/5)	57.14% (4/7)	50.00% (3/6)
*χ^2^*	0.000	0.533	0.000	0.283	0.000	1.697	1.778
*P*	1.000	0.465	1.000	0.595	1.000	0.193	0.182

## DISCUSSION

We assessed the accuracy of VEEG combined with MRI-DTI to diagnose patients with epilepsy. Our results showed that the accuracy of the combined diagnostic techniques for the seizure type and the epileptic focus location were 98.18% and 100.0%, higher than the separate accuracy of each MRI-DTI (85.45%, 85.45%) and VEEG (81.82%, 83.64%) alone. Moreover, MRI-DTI combined with VEEG technology can detect the brain tissue damage types, such as abnormal neuronal migration, tuberous sclerosis, abnormal signals, brain dysplasia, brain malformations, encephalomalacia, and cerebral atrophy, which is basically consistent with Tao et al.^18^ has reported the effectiveness of VEEG combined with MRI-DTI in diagnosing and localizing focal cortical dysplasia, but we also found the the effectiveness of VEEG combined with MRI-DTI in diagnosing abnormal neuronal migration, tuberous sclerosis, abnormal signals, brain malformations, encephalomalacia, and cerebral atrophy.

VEEG is commonly used for epilepsy assessments; and, during VEEG monitoring, an operator can record the onset of the disease. However, the overall effect of VEEG application alone is unsatisfactory.^19^,^20^ Compared to VEEG examination, brain MRI-DTI examination provides higher resolution, can avoid bone artifact interferences, and can more clearly present small lesions in the posterior fossa, middle skull, brainstem, and other areas (especially white matter lesions).^21^,^22^ Zacharia et al^23^ showed that patients with epilepsy exhibit abnormally high signal intensity in the hippocampus on T2 weighted MRI images, and that this high signal site of the hippocampus undergoes atrophy. Gonen et al^24^ confirmed that early T2WI MRI changes in high field strength and clinical MRI scanning can be used to predict the onset of secondary epilepsy, indicating that cranial MRI provides very valuable imaging information on brain lesions and prognosis assessments of status epilepticus, and this information can be used by clinicians to timely and effectively formulate diagnoses and treatment plans. Opheim et al^25^ explored the application value of MRI technology in the assessment of brain function in patients with epilepsy, and their results confirmed that MRI is a valuable tool for the assessment of brain function in patients with epilepsy, providing an important reference basis for the development of disease diagnoses, and treatment and health management plans. The results of our study confirm the high value of MRI for the diagnosis of epilepsy as reported in those studies mentioned.

Ikemoto et al^26^ showed that synchronous electroencephalography functional magnetic resonance imaging (EEG fMRI), a unique non-invasive method to assess epileptic activity, can find the deep sources of epileptic activity that cannot be detected by scalp electroencephalography or magnetoencephalogram, and the technique provides key information when considering surgical treatments or electrode implantation. Sadjadi et al^27^ showed that combined fMRI and EEG can be used to plan surgical operations in patients with drug-resistant epilepsy.

The development of MRI scanner hardware, sequences, and data post-processing has led to progress in the field of epilepsy, where ultra-high field MRI can provide high-quality datasets that can be input into post-processing programs to extract pathological features of structural or functional anatomy in patients with epilepsy.^28^ Zhu et al^29^ have shown that patients with epilepsy present varying degrees of functional and structural brain changes, and MRI examination can clarify the changes and dysfunction of local brain activity in the frontal lobe and limbic system. Saddiki et al^30^ demonstrated that MRI examinations can be applied to identify differences in speech and visual learning ability among patients with left temporal lobe epilepsy, right temporal lobe epilepsy, and extratemporal lobe epilepsy; and, abnormal bilateral hippocampal activation can be detected. In all, the evidence suggests that combining MRI-DTI with VEEG results in a high diagnostic value for the management of epilepsy.^27^-^30^

### Limitations

This was a retrospective study with a small sample size, which may have selection bias and information bias. Our findings need to be confirmed by the results of clinical prospective cohort studies. Furthermore, the levels of expertise of the radiologists may affect the results.

## CONCLUSION

The application of MRI-DTI combined with VEEG to diagnose patients with epilepsy allows for the identification of abnormal structural changes in brain and the location of lesions. Combining both approaches can improve the diagnostic accuracy of each technique alone and provide a reference for the formulation and adjustment of disease management plans.

### Authors’ contributions:

**LW:** Conceived and designed the study.

**MF**, **BZ**, **XW** and **XL:** Collected the data and performed the analysis.

**LW:** Was involved in the writing of the manuscript and is responsible for the integrity of the study.

All authors have read and approved the final manuscript.
